# Current and Future Landscape in Genetic Therapies for Leber Hereditary Optic Neuropathy

**DOI:** 10.3390/cells12152013

**Published:** 2023-08-07

**Authors:** Hoda Shamsnajafabadi, Robert E. MacLaren, Jasmina Cehajic-Kapetanovic

**Affiliations:** 1 Nuffield Laboratory of Ophthalmology, Department of Clinical Neurosciences, Oxford University, Oxford OX3 9DU, UK; 2 Oxford Eye Hospital, Oxford University NHS Foundation Trust, John Radcliffe Hospital, Oxford OX3 9DU, UK

**Keywords:** leber hereditary optic neuropathy, LHON, NADH dehydrogenase, retinal ganglion cells, gene therapy, mitochondrial inheritance, idebenone

## Abstract

Leber hereditary optic neuropathy (LHON) is the most common primary mitochondrial genetic disease that causes blindness in young adults. Over 50 inherited mitochondrial DNA (mtDNA) variations are associated with LHON; however, more than 95% of cases are caused by one of three missense variations (m.11778 G > A, m.3460 G > A, and m.14484 T > C) encoding for subunits ND4, ND1, and ND6 of the respiration complex I, respectively. These variants remain silent until further and currently poorly understood genetic and environmental factors precipitate the visual loss. The clinical course that ensues is variable, and a convincing treatment for LHON has yet to emerge. In 2015, an antioxidant idebenone (Raxone) received European marketing authorisation to treat visual impairment in patients with LHON, and since then it was introduced into clinical practice in several European countries. Alternative therapeutic strategies, including gene therapy and gene editing, antioxidant and neurotrophic agents, mitochondrial biogenesis, mitochondrial replacement, and stem cell therapies are being investigated in how effective they might be in altering the course of the disease. Allotopic gene therapies are in the most advanced stage of development (phase III clinical trials) whilst most other agents are in phase I or II trials or at pre-clinical stages. This manuscript discusses the phenotype and genotype of the LHON disease with complexities and peculiarities such as incomplete penetrance and gender bias, which have challenged the therapies in development emphasising the most recent use of gene therapy. Furthermore, we review the latest results of the three clinical trials based on adeno-associated viral (AAV) vector-mediated delivery of NADH dehydrogenase subunit 4 (ND4) with mitochondrial targeting sequence, highlighting the differences in the vector design and the rationale behind their use in the allotopic transfer.

## 1. Introduction

Leber hereditary optic neuropathy (LHON) is the most common mitochondrially inherited disease that causes visual impairment. It affects ~1 in 30,000 to 1 in 50,000 individuals [1,2]. The symptoms of the disease were first described by the German ophthalmologist Theodor Leber in 1871 [3] and linked to mitochondrial DNA (mtDNA) variations by Wallace in 1988 [4] which led to mitochondrial dysfunction and ultimately cell death.

The hallmark of the disease is the selective vulnerability and premature degeneration of retinal ganglion cells (RGCs) leading to optic nerve atrophy and profound vision loss. The painless loss of vision occurs in children and young adults, and it typically affects the central visual field in one eye, followed by the loss of vision in the other eye within weeks to months. Sometimes the loss of vision is simultaneous in both eyes. There is a possibility that LHON patient can recover within a year, but this largely depends on the age of onset and mitochondrial genotype, and most patients with LHON become and remain legally blind, significantly impacting their quality of life [5,6,7].

Even though over 50 variations in mtDNA are associated with LHON, 95% of cases are caused by one of three mtDNA missense changes: m.11778 G > A (60%) [4,8], m.14484 T > C (14%) [9,10,11], and m.3460 G > A (13%) [12,13] encoding for nicotinamide adenine dinucleotide ND4, ND1, and ND6 subunits of the respiration complex I, respectively [9,14]. Due to the variable partitioning of the different mitochondrial haplotypes in each ovum, the penetrance is highly variable within and among families, making the condition difficult to predict [15]. Only a proportion of those that carry the mutation will thus develop the disease, with estimates for 50% of male and 10% of female carriers becoming affected [16].

Currently, there is no cure for LHON. Management of patients is limited to the best supportive care. Treatments based on antioxidant therapies such as idebenone, (licenced by EMA and available in some European countries) although promising, have shown limited overall efficacy dependent on disease stage and age of onset [17]. Advanced therapeutic modalities such as allotopic gene therapy, gene editing, mitochondrial biogenesis, mitochondrial replacement therapy, and stem cell therapy have been investigated, but an effective treatment for LHON is yet to emerge [18,19,20,21]. Researchers are faced with many difficulties when developing therapies for mitochondrial disease, the key challenges being our suboptimal approaches to deliver therapeutics including nucleic acids into the mitochondria and the lack of pre-clinical model systems. The nucleic acids are hydrophilic so even small naked DNA sequences do not cross the mitochondrial double membranes when unaided. Chemical approaches destabilise membranes with potential cytotoxic side effects [22]. Biological strategies to target mtDNA in mitochondrial intracellular space have, therefore, relied on mitochondrial targeting sequences (MTSs) added to therapeutic constructs via AAV capsids [23], mitochondrial proteins [24], or mRNA [25] to push them across the mitochondrial membranes. This sophisticated allotopic gene transfer where a mitochondrial gene is recoded into the nuclear codon, thereby allowing for protein expression in the cytoplasm and its subsequent transfer into the mitochondria [24,26,27,28] or a direct transfer of nuclear-transcribed mRNA to mitochondrial membrane for protein translation in the mitochondria [21,25,29,30], has formed the basis of three current LHON gene therapy trials [31,32,33,34,35,36,37,38,39,40,41,42,43,44].

These clinical trials are underway in Europe, the United States, and China and each involves the intravitreal injection of an AAV2 vector delivering normal copies of the ND4 complex I subunit in patients with LHON carrying m.11778 G > A variant. In this manuscript, we review the clinical phenotypes and complex molecular genetics of LHON disease, highlighting the peculiarities and challenges faced when developing new therapies with emphasis on the use of gene therapy in recent years.

## 2. Clinical Presentation of LHON

**Figure 1:**
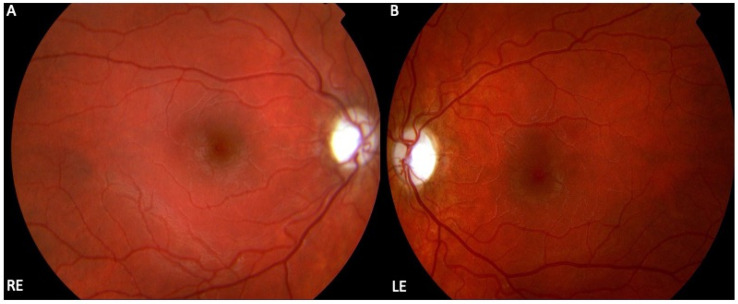
Clinical characteristics of Leber hereditary optic neuropathy (LHON). Colour fundus photographs of a 13-year-old girl with a loss of vision in both eyes (best corrected visual acuity 6/36 Snellen, right eye and left eye) showing bilaterally pale optic nerve discs (right eye (**A**), left eye (**B**)) and atrophy in the temporal part which is associated with the loss of papillo-macular bundle fibres and their associated astrocytes.

The typical LHON clinical presentation is the subacute onset of painless loss of central vision with sequential involvement in both eyes in 75% of cases or simultaneous presentation in 25% [16,45]. The time interval between the onset of sequential vision loss varies from a few weeks to a few months, with a median delay of 6 to 8 weeks. Since the majority of patients with LHON have bilateral involvement within one year (97% for m.11778 G > A variant) if the patient presents with unilateral optic neuropathy for more than one year, the patient is very unlikely to have the disease [6,46]. As the disease progresses, LHON goes through three phases: pre-symptomatic, acute, and atrophic. In the pre-symptomatic stage, patients typically do not present with obvious clinical symptoms but are diagnosed during family screenings. In the acute phase, the nerve fiber layer is swollen around the optic disc and circumpapillary telangiectatic microangiopathy affects visual acuity and colour vision. This stage ends with the disappearance of microangiopathy and the bundling of the first papillo-macular nerve fibers. As the disease progresses into the atrophic stage, optic nerve atrophy and thinning of the retinal nerve fiber layer become the pathological hallmark of the disease (Figure 1).

Although traditionally considered a disease that mostly affects young males, recent epidemiology studies show that LHON disease affects males and females of all ages with a 3:1 male-to-female ratio [7]. Females carrying an LHON pathogenic variant are, therefore, also at risk of losing vision, with the median age of onset of symptoms being slightly later at 30 years compared to 20 years for males [7]. Two major prognostic factors of visual outcome are the mitochondrial genotype and age at the onset of vision loss. There is, therefore, a possibility that visual field loss and colour vision can recover within a year, dependent on the specific variant of the mtDNA and the age of symptom onset. The reported rates vary depending on the criteria used to assess the rate of recovery. Overall, LHON patients harbouring m.11778 G > A variant represent the worst visual prognosis with only 4% visual recovery, although a recent prospective trial noted spontaneous recovery of visual acuity of 3 lines or more in 18% of patients occurring months to years after the initial loss of vision [47]. A total of 22% of patients carrying m.3460 G > A variant and 37% to 65% of patients with m.14484 T > C variant, and the best visual prognosis, recover some of their vision spontaneously [11,48] (Table 1). In addition, patients are more likely to recover when they are under 20 years of age [49], especially under 12 years of age, at the onset of the disease [50].

## 3. Molecular Genetics of LHON

**Figure 2:**
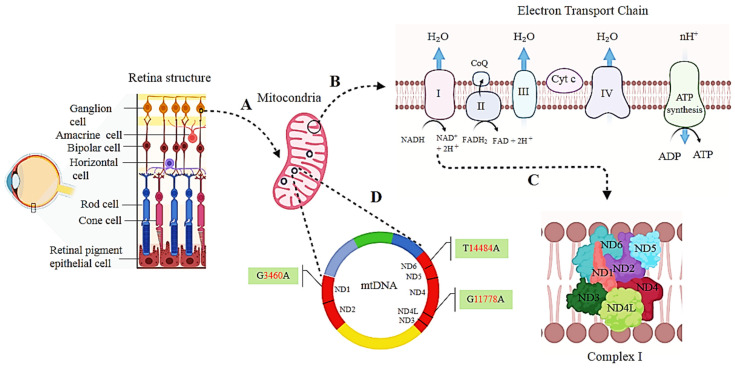
LHON is associated with mitochondrial DNA (mtDNA) variations leading to selective loss of retinal ganglion cells (RGCs). RGCs are high energy-demanding cells that prepare their energy using mitochondrial electron transport chains (**A**) where electrons are shuttled from complex I to IV in the mitochondrial respiratory chain to provide energy by transferring protons across complex V (**B**). Complex I is composed of ND1, ND2, ND3, ND4, ND4L, ND5, and ND6 subunits (**C**) encoded by mtDNA (**D**). The three most common primary mtDNA missense variants m.11778 G > A, m.14484 T > C, and m.3460 G > A in ND1, ND4, and ND6 subunits, respectively, lead to LHON disease. The figure was created using BioRender (www.biorender.com).

In order to develop new therapeutic approaches, it is essential to fully understand both the pathogenic and natural compensatory mechanisms within the retinal ganglion cells (RGCs)—the primary cell type affected in LHON (Figure 2). A major challenge in understanding the mechanisms linking LHON variants to the selective neurodegeneration of RGCs is the lack of access to human tissue to study these cells and the limitations of existing animal models. In the retina, RGCs are a type of neuron located near the inner part of the retina which carry visual signals from photoreceptors to the brain in the form of action potentials. Approximately 1.2 million axons from RGCs run in the retinal nerve fibre layer and converge at the optic nerve head passing through the lamina cribrosa to form the optic nerve. These axons need high energy and mitochondria concentration to transmit visual information from the retina to the brain. RGCs produce adenosine triphosphate (ATP) due to oxidative phosphorylation of nutrients within the mitochondria. Several enzymes are involved in oxidative phosphorylation, including NADH dehydrogenase (complex I), Succinate dehydrogenase (complex II), Cytochrome b-c1 complex (complex III), Cytochrome c oxidase (complex IV), and ATP synthase. In the mitochondrial respiratory chain, electrons are shuttled from complex I to IV and provide energy by transferring protons across the membrane. As a protons channel, complex V conserves energy by catalysing ATP synthesis from adenosine diphosphate and inorganic phosphate. The mitochondrial respiratory chain comprises ~92 nuclear and mitochondrial DNA-encoded protein subunits. The presence of any related pathogenic variants mutation results in a reduction in ATP synthesis and increased production of reactive oxidative species [51]. A combination of these factors can lead to energy failure and cell death (Figure 2). In addition to high energy demand, the selective vulnerability to energy failure of RGCs could potentially be due to their narrow axons (especially for P-type RGCs at the papillo-macular bundle) and the lack of myelination necessary for retinal transparency and light penetration to photoreceptors.

A mitochondrial genome consists of multiple copies of circular 16,569 base pair long mtDNA, encoding 37 genes (22 tRNAs, 2 rRNAs, and 13 subunits of the oxidative phosphorylation complex). mtDNA is characterised by a high copy number, maternal inheritance, as well as high levels of polymorphisms and variations. Variations in mtDNA are approximately 100 times higher than those in nuclear DNA, most likely due to a naked double-ring structure, insufficient mtDNA repair, and local environmental factors (possibly caused by oxidative radicals) [52]. Most variations associated with LHON are missense and found in complex I of the electron transport chain. Most of the patients with LHON (95%) are affected by either one of three primary mtDNA missense variants encoding for the respiratory chain subunits of the nicotinamide adenine dinucleotide ubiquinone- oxidoreductase (complex I) genes: 3460 G > A ND1, 11778 G > A ND4, and 14484 T > C ND6 [15]. In these patients, m.11778, m.3460, and m.14484 are present at a frequency of 69%, 13%, and 14%, respectively (Table 1). It was estimated that 22%, 24%, and 31% of the points variations in m.11778, m.14484, and m.3460 represent a symptom onset peak in 21, 15, and 21 years old, respectively [7]. Adult patients with the m.11778 G > A variant in ND4 had the worst visual outcomes, as described above [8].

The link between the LHON variants and disease development remains poorly understood. The incomplete penetrance and male prevalence indicate that a primary variant is necessary, but not sufficient to develop the disease. Thus, the three primary mtDNA variations which alter the evolutionarily conserved amino acids remain clinically silent until an unknown trigger induces dysfunction of the respiratory chain of the RGCs, resulting in vision impairment. There is no evidence of these variations in control individuals. In addition to primary LHON variations, secondary mtDNA variations still exist and express with lower frequency in unaffected control individuals. The secondary mtDNA variations co-occur with primary variations or among each other but do not change evolutionarily conserved amino acids. The occurrence of these types of variants on specific mtDNA backgrounds increases the risk of LHON. Some secondary variations, such as ND1/4206, ND5/13708, CYTB/15257, and CYTB/15812, have co-occurred with the primary variants m.11778 and m.14484 in positive European families [53]. Carriers of LHON genotypes have variable and incomplete penetrance, and only a portion of the genotype carriers (males more than females) will manifest the disease with a low annual conversion rate. LHON reported penetrance is about 50% to 60% in males and 10% to 20% in females, although, in larger cohorts, penetrance is also reported as low as 20% to 27% in males and 4% to 8% in females [1,54]. Although some carriers do not exhibit symptoms, it appears that a combination of genetic and environmental factors is strongly implicated in the onset of this condition. Disease penetrance is influenced by many genetic factors, including secondary mtDNA variations, the mtDNA copy number, and nuclear genes associated with correct maintenance and expression of mtDNA, heteroplasmy, and mtDNA haplogroups [55]. RGCs contain many mitochondria and, therefore, many copies of mtDNA. The levels of mutated mtDNA can vary from 0 to 100% and co-exist with wild-type mtDNA (heteroplasmy), or all mtDNA are either variant or wild-type (homoplasmy). In LHON, over 85% of the cases are homoplasmic states. Furthermore, LHON pathogenic variant carriers are at an increased risk of vision loss due to environmental factors such as tobacco smoking, excessive alcohol consumption, and toxic drug exposure [56]. The age factor is a major confounding factor contributing to the emergence of symptoms in older, asymptomatic individuals [57].

## 4. Treatment Modalities for LHON Disease

**Figure 3:**
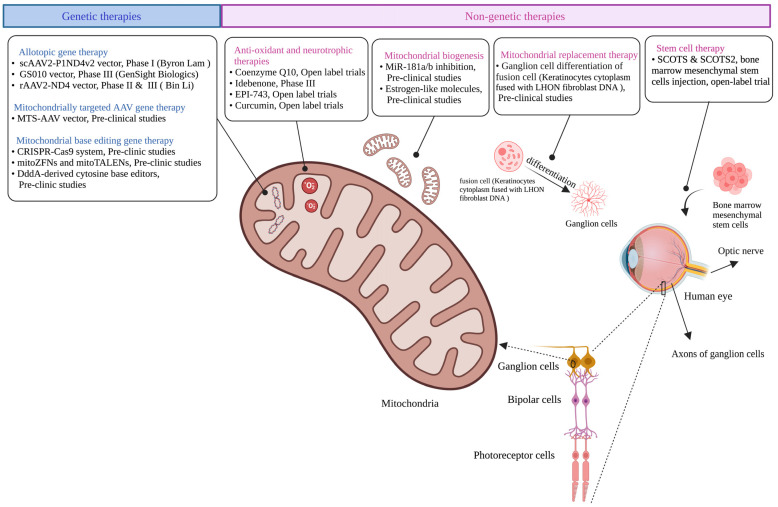
Schematic presentation of different therapeutic approaches in LHON. Current therapeutic strategies for LHON include genetic therapies, antioxidant and neurotrophic therapies, mitochondrial biogenesis, mitochondrial replacement therapy, and stem cell therapy. The figure was created using BioRender (www.biorender.com).

Several therapeutic strategies are in development to manage LHON, including genetic therapies, antioxidant and neurotrophic therapies, mitochondrial biogenesis, mitochondrial replacement therapy, and stem cell therapy (Figure 3). This review focuses on gene therapy for LHON, including the latest updates. In addition, we briefly discuss other potential therapies in development.

### 4.1. Genetic Therapies in LHON

Using gene therapy to replace the defective gene with the normal wild-type gene is an appealing treatment option for LHON, given that the RGC layer of the retina is relatively accessible for the delivery of corrected genes compared to other human tissue [58]. Among the potential gene therapies for mtDNA variations, allotopic gene therapy and mtDNA gene editing are the most promising approaches.

#### 4.1.1. Allotopic Gene Therapy

**Figure 4:**
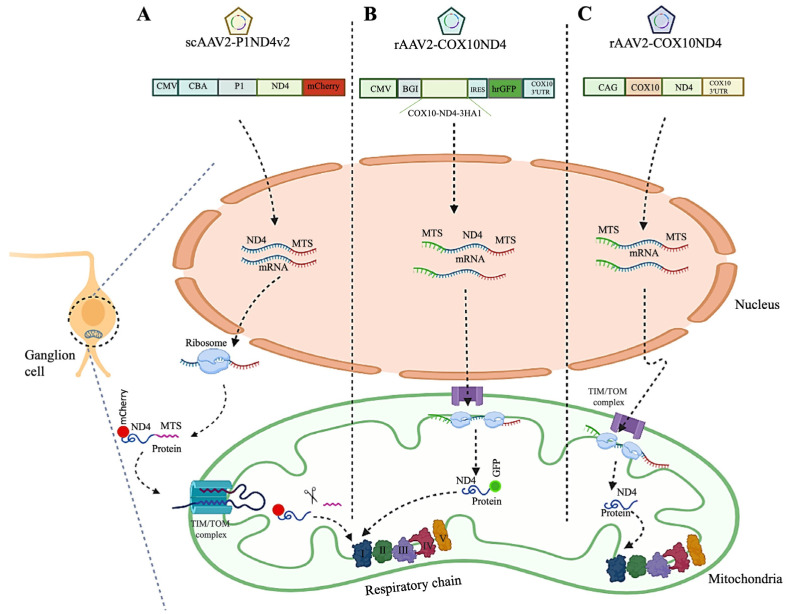
Allotopic gene therapy approaches for LHON disease. (**A**) The first allotopic gene therapy was performed using the scAAV2-P1ND4v2 viral vector carrying wild-type ND4 with the MTS nucleotide sequence from the ATP1 gene (P1). Following the delivery of the vector particles, genetic cargo is recorded in the nucleus and transcribed into mRNA which is transported into the cytoplasm for cytosolic ribosomal translation of the product protein containing the P1 MTS (P1-ND4). ND4-P1 is subsequently actively transported into mitochondria and integrated into complex I of mitochondrial respiratory chain (**B**) Second allotopic gene therapy was performed with rAAV2-COX10ND4 viral vector. This trial introduced a new MTS that allowed efficient allotopic expression of the human COX10ND4-cDNA in the nucleus followed by direct shuttling of mRNA into the outer membrane of the mitochondria. In turn, mitochondrial ribosomes translate mRNA into wild-type ND4 protein. (**C**) The third approach, also based on the AAV2-ND4 and the COX10 MTS (rAAV2-COX10ND4), allowed allotopic ND4 protein expression in the mitochondria in a similar manner to approach 2. The figure was created using BioRender (www.biorender.com).

Allotopic gene expression was developed to overcome the natural barrier to transporting molecules into the mitochondria—the mitochondrial double membrane. Applying this technique, mitochondrial genes are inserted into the nucleus, followed by the import of the resulting protein or mRNA with mitochondrial targeting sequence from the cytoplasm into the mitochondria. The efficiency of this method depends on the MTS arrangements and the protein assembly within the oxidative phosphorylation complexes (Figure 4).

##### Pre-Clinical Allotopic Gene Therapy Studies

In 2002, the first allotopic gene therapy approach was examined in human m.11778 LHON hybrids by a group of researchers led by the late John Guy and colleagues. As ND4 genes are expressed in the nucleus, a nuclear-encoded ND4 version should be prepared by converting the “non-standard” codons of the mitochondrial genetic system into the universal genetic code. Furthermore, the ND4 gene must contain enhancers, promoters, mitochondrial targeting sequences, polyadenylation signals, and a reporter gene. To direct the import of the recoded ND4 protein into the mitochondria from the cytoplasm, an MTS specifying the N-terminal region of either the P1 isoform of subunit c of human ATP synthase (ATPc) containing the entire 61 amino acid MTS plus the first five amino acids of the mature P1 polypeptide or the Aldh containing the first 19 amino acids MTS were used. Transduction of LHON cybrids with AAV-ND4 viral vectors resulted in effective expression of AAV-CMV-CBA-P1-ND4-FLAG and AAV-CMV-CBA-Aldh-ND4-GFP viral vector in the cytoplasm and import fusion protein into mitochondria. ATP synthesis was increased threefold in AAV-CMV-CBA-P1-ND4-FLAG complemented cybrids, while no effect was observed in AAV-CMV-CBA-Aldh-ND4-GFP treated cells. The first demonstration of allotopic respiration in vitro opened new prospects for future LHON gene therapy [26]. They produced the LHON mouse model by allotopic expression of the variant human R340H ND4 subunit of complex I in 2007, eliciting the hallmarks of human mitochondrial disease. According to their report in 2009, intravitreal injection of AAV2-CMV-CBA-P1-ND4-FLAG vector into LHON mouse model induced efficient expression in ganglion cells, while it did not induce the loss of RGCs, ATP synthesis, or PERG amplitude [24,27]. To follow up, they examined the safety and effects of scAAV2 (Y444,500,730F) -CMV-CBA-P1-ND4-FLAG viral vector intraocular injection into the eyes of mice, ex vivo human eye, rodents, and primates. They successfully reported that the human ND4 protein is expressed in almost all mouse RGCs and is integrated into complex I. In rodent models treated with this technique, vision is preserved, ATP synthesis is restored, and RGCs and optic nerve axons are prevented from being lost. In treated primates, a 3-month follow-up shows no adverse reactions. Ex vivo injection of ND4 into the human eye resulted in the expression of the protein in most retinal ganglion cells. Based on the results of this study, clinical testing followed for patients with the mitochondrial DNA variation 11778G > A [28].

In addition to targeting the ND4 protein to mitochondria, this group also worked on redirecting the AAV virions (carrying the ND4 gene) toward the mitochondria by the addition of the 23-aa cytochrome oxidase subunit 8 (COX8) sequence to the N terminus AAV2 capsid (COX8 MTS AAV capsid). The result demonstrated expression of the MTS AAV-delivered ND4 with the rescue of ATP synthesis in cultured LHON 11778G > A cybrids cells and visual loss and optic atrophy in rodents for almost their entire laboratory life span [59]. Using the same principle of MTS-tagged AAVs, the group recently reported that the MTS-AAV vector carrying wild-type human *ND1* restored mitochondrial respiratory complex I activity and ATP synthesis rate and protected RGCs and their axons from dysfunction and degeneration [23].

In 2007, another group of researchers led by Sahel and colleagues developed allotopic expression of *ND4* based on redirecting the mRNA to the mitochondria, in contrast to Guy’s group approach where expressed ND4 protein is redirected. Through this MTS, derived from mitochondrial protein *COX10*, a modified complementary DNA (cDNA) encoding the human wild-type mitochondrial *ND4* gene was expressed allotopically in the nucleus by shuttling mRNA directly from the nucleus to the outer membrane of the mitochondria [25]. As an MTS (21 residues N-terminal and seven additional residues that ensure proper MTS cleavage (MAASPHTLSSRLLTGCVGGSVWYLERRT)), COX10 was introduced to a gene therapy vector in m.11778 LHON fibroblasts cells. As a result of transfection with the CMV-COX10-ND4-IRES-COX10 3′UTR-FLAG vector, the cells exhibited stably expressed ND4 protein and RNA, efficient mitochondrial translocation, as well as a significant restoration of oxidative phosphorylation activity [29,60]. The study was further extended by producing and injecting the pAAV-COX10-ND4-IRES-hrGFP viral vector into the rat model’s vitreous body. Based on the findings of this study, the expression of human ND4 prevented the loss of RGCs and the impairment of visual function in the LHON rat model [21]. The safety and efficacy of this vector were confirmed in the LHON rat model by intravitreal injections of AAV2.2-CMV-beta globin intron-ND4-IRES-3HA1-hrGFP-COX10 3′UTR viral vector in 2015. In this study, human ND4 expression was demonstrated not to be harmful, with assembly in the respiratory chain complex I, and was found to significantly inhibit the degeneration of RGCs [25].

In 2013, a team of scientists led by Li and colleagues developed another mitochondrially targeted human *ND4* based on an amino aside protein sequence. The aside amine sequence of the produced ND4 protein was precisely the same as the native mitochondrial protein. An AAV2neo-CAG-COX10-ND4-COX103′UTR vector was designed based on the newly targeted gene and without a reporter sequence. The ND4 gene was confirmed to be translocated to the mitochondria in transduced HEK293 cells [30].

##### Clinical Allotopic Gene Therapy Trials

**Figure 5:**
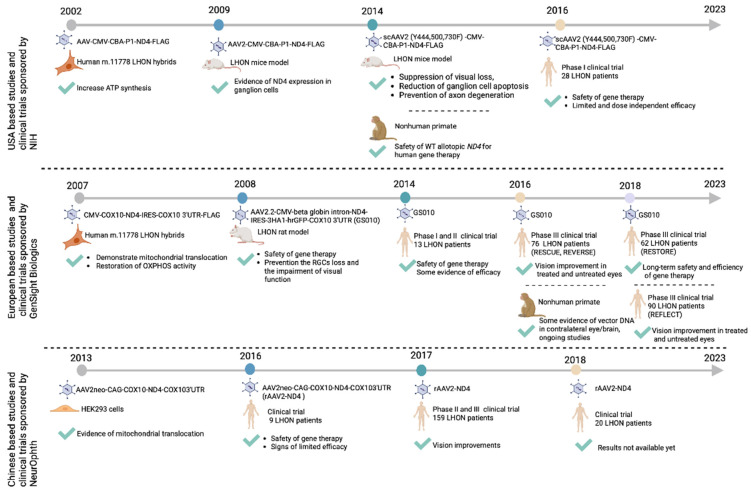
The development of LHON gene therapy from bench to bedside. Following proof-of-concept studies and having demonstrated safety and efficacy in pre-clinical models, three major clinical trials were launched, each aimed at testing safety and efficacy of gene supplementation of wild-type *ND4* in patients with mutant 11778 G > A *ND4.* The figure was created using BioRender (www.biorender.com).

The results generated from the pre-clinical allotopic gene therapy studies formed the basis for launching the three major LHON clinical trials in the USA (NCT02161380, sponsored by NIH), Europe (NCT02064569, sponsored by GenSight Biologics), and China (NCT01267422, sponsored by NeurOphth) (Figure 5). Each trial was designed to supplement wild-type human *ND4* in patients carrying the most common LHON variant, the m.11778 G > A *ND4* variant.

An open-label dose escalation study (NCT02161380) was initiated in 2014 at the University of Miami, to demonstrate the safety of low and medium doses of scAAV2(Y444,500,730F)-P1ND4v2 viral vector for the treatment of LHON patients. The drug was injected intravitreally into one eye in five participants with vision loss secondary to G11778A LHON. A 90 to 180-day follow-up period was conducted on the treated participants, and ocular and systemic safety assessments were performed, as well as the visual structure and function examination. This initial phase I study revealed no serious safety concerns [34]. This trial was continued with intravitreal injection of vector into one eye of six participants with a chronic bilateral visual loss greater than 12 months (Group 1), six participants with bilateral visual loss less than 12 months (Group 2), and two participants with unilateral visual loss (Group 3) with a minimum of 12 months of follow-up. During the 12-month evaluation, it was confirmed that the average improvement in the injected eye was 0.24 logMAR, more significant than the 0.09 logMAR improvement observed in the fellow eyes. The average temporal RNFL thickness had not changed from baseline in the treated eyes, whereas it had reduced significantly in the fellow eyes 12 months after injection [31]. The longitudinal trial was completed in a total of 28 patients (5 females) after unilateral intravitreal injection of low, medium, high, and higher vector doses to patients with G11778A LHON and chronic bilateral visual loss > 12 months (Group 1, *n* = 11, worse eye), acute bilateral visual loss < 12 months (Group 2, *n* = 9, worse eye), or unilateral visual loss (Group 3, *n* = 8, better eye). The study result represents that incident uveitis (8 of 28, 29%) as a vector-related adverse event was related to vector dose and occurred in 71% of higher-dose eyes, and 14% of low, medium, or high-dose eyes. Improvements of ≥15-letter best-corrected visual acuity (BCVA) occurred in some treated and fellow eyes of Groups 1 and 2 and some surrogate study and fellow eyes of natural history subjects. Despite treatment, all study eyes (BCVA ≥ 20/40) in Group 3 lost ≥15 letters within the first year. The researchers concluded that the treatment efficacy, if any, is likely to be limited and dose independent. In addition, asymptomatic eyes with unilateral vision loss may be beyond the window of opportunity for neuroprotection given the reduced thickness of the ganglion cell layer and impaired pattern electroretinogram. Randomisation of patients to a control group that does not receive the vector in either eye would help to assess the treatment effect given confounders of subclinical disease and variable natural history [35].

In parallel, a Europe-based trial sponsored by GenSight Biologics (NCT02064569) was launched in 2014 to evaluate the safety of their vector AAV2.2-COX10ND4 (GS010). A single intravitreal injection of the vector was given to the worse-seeing eye of 19 LHON patients carrying the m.11778 variation. Initial follow-up results indicated no serious adverse events related to the treatment. Six of fourteen subjects with treated eyes had a clinically significant improvement in BCVA two years after injection [36]. A five-year follow-up revealed no serious adverse events related to the treatment or procedure. A clinically significant improvement in BCVA was observed in six of fourteen subjects at week 96 (similar proportions were observed at weeks 48 and 78) [37]. In 2016, two phase III clinical trials started to evaluate the clinical efficacy of GS010 in 76 LHON patients affected for up to 6 months (RESCUE, NCT02652767) and more than 6 months to 12 months (REVERSE, NCT02652780) by m.11778 variation in France, Germany, Italy, the United Kingdom, and the United States. Patients received a single dose of lenadogene nolparvovec (GS010) via a single intravitreal injection containing 9 × 10^10^ viral genomes in 90 μL in one of their randomly selected eyes [38]. The efficacy analysis of the RESCUE trial (multi-centre, randomised, double-masked, sham-controlled) included 38 subjects with a mean age of 36.8 years (82% male). Treatment started around 3.6 months and 3.9 months after the disease onset in the rAAV2/2-ND4 treated and sham-treated eyes, respectively. At week 48, the difference in BCVA from baseline between rAAV2/2-ND4 treated and sham-treated eyes were 0.01 logMAR, while it was 0.03 logMAR at week 96 [38]. The REVERSE trial (enrolled a total of 37 subjects) result showed that 25 subjects (68%) had a clinically relevant recovery in BCVA from baseline in at least one eye, and 29 subjects (78%) had an improvement in vision in both eyes (treated and sham). The difference in the change in BCVA between rAAV2/2-*ND4*-treated and sham-treated eyes at week 48 was −0.007 logMAR. Meanwhile, at week 96, there was an improvement in BCVA of −0.308 (0.068) logMAR, equivalent to a gain of 15 Early Treatment Diabetic Retinopathy Study (ET-DRS) letters (*p* < 0.0001 for change from baseline). The mean change from the baseline in BCVA increased continuously and bilaterally over the 96 weeks after treatment.

The possible mechanisms underlying the improvement in vision in the contralateral untreated eye were further investigated in nonhuman primates (NHPs). Three months after unilateral intravitreal injection of rAAV2/2-*ND4* in the NHP right eye, rAAV2/2-*ND4* DNA was detected in all the tissue and fluid samples tested for the three animals, including an anterior segment of the contralateral eye, retina, optic nerve, optic tract, lateral geniculate nucleus, and optic chiasm. In contrast, the contralateral visual cortex was not infected with the vector [39]. Further experiments are underway to explore this unexpected contralateral effect observed in clinical trials.

RESCUE subjects were treated on average 16 weeks after the onset of vision loss, whereas REVERSE subjects were treated 39 weeks after the onset of vision loss. On average, recovery of BCVA was observed 24 weeks after treatment in the RESCUE trial and 12 weeks after treatment in the REVERSE trial. Contrary to expectations, despite earlier treatment in RESCUE, visual outcomes at 96 weeks were less favourable than those among subjects treated at later stages of disease in REVERSE [40]. In 2018, the RESCUE or REVERSE long-term follow-up study assessed the safety and efficacy of GS010 in 62 (31 from REVERSE and 31 from RESCUE) patients with LHON five years after injection (RESTORE, NCT034061104). According to the 4-year data, objective visual acuity improvements are sustained and are associated with improvements in functional quality of life without any long-term safety concerns [33].

Another phase III, the global, multi-centre randomised, double-masked trial, was initiated in 2018 to evaluate the efficacy and safety of intravitreal injections of 9 × 10^10^ GS010 viral in 90 μL in 98 LHON patients up to 1 year from vision loss onset (REFLECT, NCT03293524). An intravitreal injection was administered in the first affected eye in all affected patients. Meanwhile, the second affected eye was randomised to either a second injection or a placebo injection. A bilateral treatment was administered to 48 patients, and a unilateral treatment to 50 patients. After two years, the mean BCVA in treated eyes was statistically significantly better than baseline, whereas improvement from baseline was not statistically significant in placebo-treated eyes. The results indicate a sustained treatment effect for all subjects, with the maximum improvement among bilaterally treated patients (https://www.gensight-biologics.com, accessed on 14 December 2021). GS010 currently remains an investigational compound and has not been registered in any country at this stage. A marketing authorization application is currently under review by the European Medicines Agency (EMA). GenSight also aims to resume its interactions with FDA to secure a regulatory pathway in the United States.

The third pre-clinical vector (pAAV2neo-COX10-ND4-COX103′UTR, or rAAV2-ND4) was taken into an investigator-led clinical trial (NCT01267422) in China. Nine patients were administered with the vector by intravitreal injection to one eye and then followed for 9 months During the 9-month follow-up period, no patients had local or systemic adverse events related to the vector, and the visual acuity (VA) of six patients improved by at least 0.3 logMAR. In these six patients, the VF was enlarged, but the RNFL remained relatively stable [41]. This trial’s long-term results (3 years) confirmed the safety of intravitreal injection of rAAV2-ND4 for treating LHON. A significant improvement in BCVA, VF, and visual evoked potential (VEP) was also observed in treated eyes [61]. In 2017, a phase II and phase III clinical trial (NCT03153293) was initiated. The patients were treated intravitreally with a single injection of rAAV2-ND4 viral vector, with a 1 × 10^10^ virus dose in 0.05 mL. According to the results, higher baseline VFI, smaller baseline VF mean deviation, and higher baseline BCVA were associated with better VA outcomes. Long-term follow-up of these patients confirmed the strong association of age, the period between onset and treatment, and pre-treatment baseline BCVA with rapid and significant improvement in the VA of the injected eye [42,43]. It should also be noted that the severity of VF defects appeared independent of age, and it progressed within 6 months of disease onset remaining stable after 9 months. The differences in visual function between the eyes decreased as the disease progressed [44].

A new clinical trial (NCT03428178) was initiated in 2018 to evaluate the efficacy of gene therapy in patients with LHON symptom onset within 3 months. This clinical trial recruited 20 patients with the m.11778 variation onset within 3 months, 20 between 3 to 6 months, 20 between 6 to 12 months, 20 between 12 to 24 months, 20 between 24 to 60 months, and 20 over 60 months. All patients have been treated with a single injection of rAAV2-ND4 viral vector with dose 1 × 10^10^ viral 0.05 mL. A comparison will be made between the VA, VF, VEP, optical coherence tomography (OCT), electroretinograms (ERG), RNFL, and liver and kidney function in plasma before and after treatment at 1, 2, 3, 6, and 12 months. Results have not yet been published (Table 2).

#### 4.1.2. Gene Editing

In mitochondrial disease caused by heteroplasmic variations in mtDNA, the clinical manifestations depend on the variation threshold resulting in a biochemical impairment. Therefore, eliminating some variant mtDNA molecules reduces the ratio of variant to wild-type mtDNA below the threshold for disease, decreasing the biochemical defect. Several endonucleases with localisation signals have been demonstrated to be capable of reaching mitochondria, identifying pathogenic variations as targets, and selectively causing double-strand breaks in heteroplasmic mtDNA, including mitochondria-targeted restriction endonucleases (mitoREs), zinc finger nucleases (mtZFNs), and mitochondrial-targeted transcription activator-like effector nucleases (mitoTALENs) [61,62]. In addition, both mitoZFNs and mitoTALENs approaches have been shown to restore oxidative phosphorylation function in a mouse model carrying a rare heteroplasmic variation of LHON [63,64].

In mtDNA editing, CRISPR-based systems have proven to be potent tools. The process uses guide RNAs (gRNAs) corresponding to the DNA sequences to be edited and nucleases that create double-stranded DNA breaks at specific sites. It has been demonstrated that the AAV2-mediated YFP/SpCas9 editing construct achieves the most efficient *YFP* reduction in the retinal cells of *YFP* transgenic mice [65,66]. Despite the potential of CRISPR/Cas, it is restricted by the difficulty of delivering gRNA to mitochondria. Because of this reason, Hussain et al. propose a stem-loop element, called the RP-loop, as a potential enhanced mitochondrial transporter for CRISPR-Cas9 in 2021. The editing process was performed by adding a targeting guide RNA to an RP-loop and expressing the Cas9 enzyme containing mitochondrial localisation sequences. According to the results, RP-loop gRNA was colocalised with mitochondria, and transcription of the ND4 gene was significantly decreased in cells carrying 11205G. Therefore, CRISPR-Cas9 gene editing is a promising method for treating mitochondrial diseases [67]. Following the development of the CRISPR/Cas9 gene editing system, a promising candidate, mitoCas9 emerged, which targets mitochondria and is likely to be explored in future studies as a potential treatment for LHON [68]. Moreover, a CRISPR-free mitochondrial base editing with a bacterial cytidine deaminase toxin, the DddA-derived cytosine base editor (DdCBE) allowed precise manipulation of mtDNA in human cells, without the need to eliminate mtDNA copies that result from its cleavage by targeted nucleases [69].

### 4.2. Antioxidant and Neurotrophic Therapies

In the treatment of mitochondrial disorders, such as LHON, antioxidant therapies have played a crucial role. The first approach to antioxidant therapy involved nutritional supplements such as vitamins (B2, B3, B12, C, E, and folic acid), carnitine, creatine, l-arginine, dichloroacetate, or ketogenic diets. These treatments had limited and variable efficacy for patients with LHON [70]. Vitamin-like substance coenzyme Q10, or ubiquinone, was also investigated for its antioxidant properties in LHON. By ubiquinone, electrons are shuttled from complexes I and II to complex III during the mitochondrial respiratory chain. A significant improvement in visual acuity was reported after 4 months of oral treatment with increasing coenzyme Q10 [71]. Treatment with combination therapies of CoQ10 and other nutritional supplements improved plasma lactate concentrations, body composition, ankle dorsiflexion strength, and oxidative stress in patients with LHON [72]. Subsequently, a synthetic analogue of coenzyme Q10 (CoQ10), idebenone became the most studied antioxidant drug for the treatment of LHON. There is evidence that ubiquinone-treated cell lines from patients with LHON can increase ATP production, decrease ROS levels, and prevent RGC deterioration in a mouse model of LHON [73]. In a 24-week multi-centre double-blind, randomised, placebo-controlled clinical trial (NCT00747487, RHODOS), 85 LHON patients with the three common mitochondrial DNA variations were treated with idebenone (300 mg three times per day) for 6 months (Canada, Germany, and the United Kingdom). Its treatment efficiency is controversial; effective in some patients but failed in others [74]. As a result of idebenone treatment, patients at imminent risk of further vision loss were able to maintain tritan colour vision and prevent any further loss of colour vision [75]. Following the conclusion of the previous trial, 39 LHON patients treated with idebenone were enrolled in a follow-up trial 30 months after discontinuation of the treatment (NCT01421381, RHODOS-OFU). Even after the drug was discontinued, RHODOS-OFU results indicate that VA continued to improve [76].

The results of a parallel study on 103 LHON patients revealed that earlier visual improvement was associated with younger age, the presence of the m.14484 variation, prompt initiation of the treatment, and a longer duration of the therapy [77].

In 2015, idebenone (Raxone^®^) was approved by the European Medicines Agency (EMA) in the European Union for treating LHON at 900 mg/day divided into three doses. For VA improvement, idebenone treatment should be initiated during the acute phase of the disease and continue for at least one year following the onset of the visual loss. As soon as improvements cease to be observed after one year, discontinuation should be considered [78]. Further clinical trials have confirmed that the duration of idebenone treatment needs to be increased to maximise its effectiveness. An open-label, multi-centre, retrospective, noncontrolled clinical trial (NCT04381091) examined the long-term VA and safety of idebenone (900 mg/day) treatment in 111 LHON patients (average treatment duration of 25.6 months). In 46% of LHON patients, clinically relevant recovery occurred between 2.5 and 26.5 months after treatment commenced, with a mean of 9.5 months. In addition to clinically relevant recovery, the magnitude of recovery increased with treatment duration.

Furthermore, idebenone treatment should be initiated early and maintained for more than 24 months to maximise its effectiveness, according to the results of this clinical trial [17]. From 2014 to 2020, a multi-centre, open-label clinical trial evaluated the efficiency of idebenone treatment on 72 Dutch LHON patients in the Netherlands. This study observed clinically relevant recovery (CRR) or clinically relevant stabilisation (CRS) in 53% and 11% of patients, respectively. Furthermore, RNFL and ganglion cell complex (GCC) thicknesses were irreversibly reduced [27]. Additional post-approval phase 4 clinical trials (NCT02774005, LEROS, and NCT02771379, PAROS) started in 2016 to examine the efficacy and safety of Raxone^®^ in the long-term treatment of LHON patients. In LEROS, visual acuity outcomes following 24 months of idebenone treatment were compared to those of an external natural history cohort. A sub-analysis according to age at symptom onset and disease phase showed that treatment benefit was stronger in patients over 18 years. Subacute eyes of patients under 18 years of age showed limited treatment effect and had high recovery rate in the natural history cohort (ARVO 2023 Abstract no. 1953). In addition, a subanalysis of the potential treatment effects according to mitochondrial mutation show positive trends towards improved functional vision in eyes with m.11778 G > A and m.14484 T > C, whereas patients with m.3460 G > A had limited treatment effect and a mild disease course in the natural history cohort (ARVO 2023 Abstract no. 1954).

In addition to idebenone, several other antioxidants, such as EPI-743, elamipretide, and curcumin have been investigated. EPI-743 is a vitamin E base drug shown to have approximately 1000 to 10,000 fold greater antioxidant activity in vitro than coenzyme Q10 or idebenone [79]. The efficacy of EPI-743 was evaluated in a small, open-label clinical trial (NCT02300753) on five genetically confirmed LHON patients (four harbouring the m.11778 variant and one having the m.3460 variant) within 90 days of the onset of disease at a dose of 100–400 mg/day for at least one year. This trial confirmed that EPI-743 arrested disease progression and reversed vision loss in four patients. Based on the results of this trial, EPI-743 was found to arrest the progression of the disease and reverse vision loss in the participants [80]. Elamipretide is another promising candidate, and it was found to reduce reactive oxygen species (ROS) and increases adenosine triphosphate (ATP) levels [81]. As a result of a prospective, randomised, double-masked clinical trial (NCT02693119) conducted on 12 LHON patients with the m.11778 mtDNA variation, elamipretide (1 drop of elamipretide 1% topical ophthalmic solution) treatment for 52 weeks was found to be safe, tolerable, and efficacious. Continuing bilateral elamipretide therapy for at least 84 weeks is associated with significant improvements in best-corrected VA and colour discrimination in patients [82].

Moreover, an antioxidant drug known as curcumin (NCT00528151) has been clinically tested in a randomized, double-blind, placebo-controlled trial (NCT00528151). The trial (250 mg twice a day) was conducted in Thailand; however, the results still need to be published.

The newest antioxidant therapy is the near-infrared light-emitting diode therapy using low-energy lasers with a red to near-infrared range (630–1000 nm) to induce energy production and antioxidant protection [83]. The near-infrared light-emitting diode treatment is evaluated in a non-randomized, interventional clinical trial (NCT01389817) using a 630 nm wavelength and 4 J/cm^2^ laser emissions to the closed eye (80 s) twice daily for 12 months. This clinical trial was conducted in 2011 involving four patients suffering from LHON, but the results are yet to become available.

Antioxidant therapies have been tested in clinical trials, but their efficacy and benefits remain unclear. Participants were instructed to maintain a regular diet, caffeine intake, fiber intake, and their normal levels of activity/exercise throughout the trial period. However, baseline differences in these participant attributes may impact the study’s outcome. Furthermore, due to the relatively small number of participants, many of these trials have included subjects with defined mutations of various genes and phenotypic disorders. Also, it is difficult to determine whether genetic abnormalities or heteroplasmy levels caused differences in efficacy responses. Due to these reasons, a double-blind, crossover trial would be beneficial since it limits the number of patients required for statistical significance and reduces the effects of heterogeneity in the starting population.

### 4.3. Mitochondrial Biogenesis and Replacement Therapies

#### 4.3.1. Mitochondrial Biogenesis

A compensatory mechanism for mitochondrial dysfunction in LHON occurs through mitobiogenesis or mitophagy. Many transcriptional activators peroxisome proliferator-activated receptor γ co-activator-1α, their regulators, and AMP-activated protein kinase include fibrates, rosiglitazone, metformin, and 5-aminoimidazole-4-carboxamide ribonucleoside are responsible for controlling mitobiogenesis [84].

Moreover, inhibiting the mammalian target of the rapamycin (mTOR) signalling pathway enhances mitophagy of dysfunctional mitochondria harbouring higher levels of variations. Rapamycin, as an mTOR inhibitor drug, induces selected mitophagy of dysfunctional mutated mitochondria and preserves the visual function in the LHON cybrid (G11778A) and Ndufs4 knockout mice model [85,86]. Several microRNAs, including miR181a/b, target Nrf1 to regulate mitochondrial biogenesis and mitophagy. In previous studies, the deactivation of MiR181a/b in LHON mouse models improved visual phenotypes. Therefore, this represents a gene-independent therapeutic target for the treatment of LHON [18].

In addition, since LHON has a gender bias, it is possible that different hormonal metabolisms between genders can affect mitochondrial function in LHON. Thus, a combination of natural estrogen-like molecules (i.e., phytoestrogens genistein, daidzein, and equol) or 17-norestradiol increased cell viability of LHON hybrid cells by reducing apoptosis, inducing mitochondrial biogenesis, and strongly reducing the levels of reactive oxygen species [87,88].

#### 4.3.2. Mitochondrial Replacement Therapy

As part of LHON treatment, preventing mtDNA variation transmission to future generations has potential therapeutic benefits. This approach involves extracting nuclear DNA from the patient’s egg or embryo and transplanting it into a donor’s cytoplasm containing wild-type mitochondrial DNA. Pronuclear transfer, spindle transfer, and polar body transfer are possible methods of mitochondrial replacement therapy. In 2017, for the first time, a cybrid approach for correcting mtDNA variations in LHON patient-derived iPSCs with homoplasmic double variations m.4160 and m.14484 was proposed. In this study, the LHON fibroblasts’ nuclear DNA was fused by wild-type keratinocytes cytoplasm. Successful mitochondrial replacement cells were isolated, expanded, and differentiated into RGC. Fused RGCs exhibited lower levels of mitochondrial superoxide than LHONs. They described the hybrid technique as a feasible approach for correcting mtDNA variations in LHON cell models, which could be applied to other mtDNA-related diseases [20]. However, this approach has been limited by active ethical considerations and unexplored long-term implications.

### 4.4. Stem Cell Therapy

The stem cell ophthalmology treatment study (SCOTS, NCT 01920867) and its follow-up study (SCOTS 2, NCT 03011541) are non-randomised, open-label efficacy trials for the treatment of retinal and optic nerve diseases by ganglion cell replacement. The mesenchymal stem cells, derived from the posterior iliac crest bone marrow, were administered through several delivery routes including retrobulbar, subtenon, intravitreal, intraocular, subretinal, and intravenous injections. The results indicated statistically significant improvements or vision stabilisations in most reported patients. Based on the results of the six LHON patients treated, 83.3% had improved their vision in both eyes within 24 months. This improvement is thought to have been achieved by transforming bone marrow mesenchymal stem cells into developed ganglion cells and transferring mitochondria and lysosomes to damaged cells [19,89]. However, claims of improvement in vision could not be verified because patients receiving the SCOTS treatment were discharged to local follow-up almost immediately after receiving their injections for which they paid up to USD 20,000 each. Hence, the SCOTS trial should not be considered seriously in any scientific debate and is only mentioned here to warn readers that its significance can be formally dismissed.

## 5. Summaries

LHON is the most common maternally inherited mitochondrial genetic disease and an important cause of blindness in children and young adults. The pathophysiology of LHON is particularly complex with peculiar specificity for RGCs, male bias, and incomplete penetrance. Research groups across the globe have made significant headways in improving our understanding of the disease mechanisms, natural history, and potential treatments. Several antioxidant therapies have been recommended, with varying outcomes, whilst idebenone became licenced by EMA in 2015 for the treatment of LHON. Post approval studies, however, have shown limited overall benefit to date with subgroup analyses showing dependence on the age at symptom onset, disease phase, and mitochondrial mutation. Currently, there are no recommendations to treat asymptomatic carriers.

Gene supplementation therapy is a logical treatment strategy for LHON, but the development of mitochondrial gene therapy has been hindered due to the difficulty in importing genes into the mitochondria and the lack of suitable pre-clinical models to carry out the studies. The development of allotopic gene transfer offered hope and its successful validation in cellular and animal models culminated in three major research groups leading multiple clinical trials. In 2015, GS010 (lenadogene nolparvovec, GenSight) received Investigational New Drug acceptance from the U.S. Food and Drug Administration (FDA) to enter phase III trials, and has indeed undergone phase III testing under three pivotal phase III trials (RESCUE, NCT02652767), (REVERSE, NCT02652780), and (REFLECT, NCT03293524). Unfortunately, despite visual improvements, beyond what would be expected based on the natural history of the disease, the overall efficacy was limited and complicated by the unexpected similar improvements in the untreated eye. This contralateral effect could be accounted for by the natural history of the disease, mechanical transfer of the viral vector DNA from one eye to the other, other potential local mediators, or simply a learning effect due to the clinical trial design and visual acuity measurements. NCT02161380 trial also suggests that if there is an efficacy effect in LHON, it is likely small and it was not uncommon in natural history patients. To ensure gene therapy efficacy was picked up, some patients would need to be randomised into a group not receiving an injection in either eye. Overall, LHON clinical trial results have contributed greatly to our understanding of LHON and provide valuable implications for future gene therapy clinical trial design and outcome measures.

## 6. Future Direction and Expert Opinion

Until now, promising progress has been seen in the gene therapy of LHON, but this treatment is yet to be approved. The pre-clinical results are encouraging, but the expression and function of ND4 in mitochondria require more evidence and more reliable assessments in pre-clinical models, which better recapitulate LHON disease. To this end, establishing induced pluripotent stem cells from patients with LHON and differentiating them into RGCs or full retinal organoids with preserved cellular microenvironments will open exciting new research opportunities and therapeutic developments. Further expression studies of mitochondrial proteins post gene therapy in an NHP model will be critical in learning the limitations of currently used vectors and the delivery method. The natural history of the LHON disease, the sequential nature of visual loss, and a varied degree of spontaneous improvement in affected individuals make the clinical trial design extremely challenging, and improvements are needed in future study designs and clinical trial endpoints, with the need to include a randomised untreated control group. Future trials should consider combination therapies, such as idebenone and gene therapy, which could result in a synergistic, complementary therapeutic effect, especially in some patient subgroups. Ongoing pre-clinical research on genome editing and CRISPR-free mitochondrial base editing has shown initial promising results and provides opportunities for further development specific to LHON. The transition of LHON genetic treatments from bench to bedside will closely relate to the collaborative effort of molecular biologists and clinicians.

## Figures and Tables

**Table 1:** Mitochondrial DNA variations associated with LHON disease and its characteristics.

mtDNA Variation/Complex I Subunit	NT Δ	AA Δ	Patients Carrying the Variant (%)	Variant Penetrance (% in Males)	Recovery of Vision (%)
m.11778 G > A/ND4	G > A	R340H	69	82	4–25
m.3460 G > A/ND1	G > A	A52T	13	40–80	22–25
m.14484 T > C/ND6	T > C	M64V	14	68	37–65

**Table 2:** Summarises clinical trials targeting LHON disease by allotopic gene therapy.

Clinical Trial Identifier and Name	Phase	Vector	Age Range (Years)	Participants Population	Primary Endpoints	Secondary Endpoints	Results
NCT02161380	I	scAAV2-P1ND4v2	15+	28	IAE	Safety, efficacy, and VA change from baseline	No serious safety problems at low and medium doses; asymptomatic uveitis in 2 patients
NCT02064569	I and II	GS010	18+	19	IAE	-	Safety and tolerability of intravitreal
REVERSE, NCT02652780	III	GS010	15+	37	VA at Week 48	VA at Week 72 and Week 96, number of eye and subject responders, GCL macular volume, RNFL temporal quadrant and papillo-macular bundle thickness, ETDRS total macular volume, VF MD and foveal threshold, contrast sensitivity, and colour vision	Improvement of BCVA in treated and sham eye
RESCUE, NCT02652767	III	GS010	15+	39	VA at Week 48	VA at Week 72 and Week 96, number of eye and subject responders, GCL macular volume, RNFL temporal quadrant and papillo-macular bundle thickness, ETDRS total macular volume, VFI/MD and foveal threshold, contrast sensitivity, and colour vision	Improvement of BCVA
RESTORE, NCT03406104	III	GS010	15+	61	IAE up to 5 years	BCVA, HVF, and OCT, responder analysis, time course of the response, GCL, VFQ-25 and SF-36-v2 up to 5 years	No serious IAE
REFLECT, NCT03293524	III	GS010	15+	90	BCVA up to 1.5 year	BCVA, responder analysis, OCT, HVF, contrast sensitivity, VFQ-25 and SF-36-v2 up to 1.5 and 2 years	Improvement of BCVA
NCT01267422	Not Applicable	rAAV2-ND4	8 years to 60 years	9	BCVA up to 3 years	IOP, neutralizing antibodies, average RNFL thickness, VFI/MD, and VFI, VEP up to 3	Improvement of BCVA, VF, and VEPs, in both treated and untreated eyes, RNFL thickness relatively unchanged
NCT03153293	II and III	rAAV2-ND4	10 years to 65 years	159	BCVA up to 1 year	VEP, RNFL, and liver function in plasma up to 1 year	Results unavailable
NCT03428178	Not Applicable	rAAV2-ND4	8 years to 60 years	120	BCVA up to 1 year	VEP, RNFL, VF MD and VFI, liver, and kidney function in plasma	Results unavailable

Abbreviations: IAE, incidence of adverse events; BCVA, best corrected visual acuity; ETDRS, early treatment diabetic retinopathy study; IOP, intraocular pressure; OCT, optical coherence tomography; RNFL, retinal nerve fiber layer; VA, visual acuity; VEP, visual evoked potentials; VF, visual field; VFI, visual field index; VFI/MD, visual field index mean deviation; GCL, ganglion cell layer; VFQ-25, Quality of Life: Visual Functioning Questionnaire 25; and SF-36-v2, Quality of Life: 36-Item Short Form Health Survey, version 2.

## Data Availability

Data sharing not applicable.
